# Authentication of EU-Authorized Edible Insect Species in Food Products by DNA Barcoding and High-Resolution Melting (HRM) Analysis

**DOI:** 10.3390/foods14050751

**Published:** 2025-02-22

**Authors:** Michaela Wildbacher, Julia Andronache, Katharina Pühringer, Stefanie Dobrovolny, Rupert Hochegger, Margit Cichna-Markl

**Affiliations:** 1 Department of Analytical Chemistry, Faculty of Chemistry, University of Vienna, Währinger Straße 38, 1090 Vienna, Austria; 2 Department for Molecular Biology and Microbiology, Institute for Food Safety Vienna, Austrian Agency for Health and Food Safety, Spargelfeldstraße 191, 1220 Vienna, Austria

**Keywords:** edible insects, food authentication, species differentiation, DNA barcoding, 16S rDNA, PCR, high-resolution melting analysis

## Abstract

The consumption of edible insects is a promising approach to meet the increasing global demand for food. Commercialization of edible insects in the EU is regulated by the Novel Food regulation. To date, the yellow mealworm (*Tenebrio molitor* larva), the migratory locust (*Locusta migratoria*), the house cricket (*Acheta domesticus*), and the buffalo worm (*Alphitobius diaperinus* larva) have been authorized in the EU for human consumption. We aimed to develop a method based on DNA barcoding and high-resolution melting (HRM) analysis for the identification and differentiation of these four EU-authorized edible insect species in food. A primer pair previously designed for DNA metabarcoding, targeting a ~200 bp sequence of mitochondrial 16S rDNA, allowed discrimination between the four insect species in highly processed food. However, house cricket and migratory locust could not unambiguously be differentiated from tropical house cricket, desert locust, superworm, cowpea weevil, and sago worm, respectively. This problem could be solved by designing primers specific for house cricket and migratory locust. By combining these primers with the insect primers, additional polymerase chain reaction (PCR) products for house cricket and migratory locust were obtained, resulting in more complex melt curves compared to the unauthorized insect species. The optimized PCR-HRM assay is a very cost-efficient screening tool for authentication of EU-authorized edible insect species in food.

## 1. Introduction

Feeding the growing world population sustainably is one of the greatest challenges of our time [1]. The rise in global population is accompanied by high levels of meat consumption [2]. Meat is an energy-dense food that provides a valuable source of proteins, essential amino acids, and micronutrients, such as iron, selenium, vitamin A, vitamin B12, and folic acid [3,4]. However, meat consumption has significant negative impacts on the environment. Meat production, in particular that of ruminants, is highly resource-intensive, requiring large tracts of land, substantial energy inputs, and significant freshwater consumption [5]. Moreover, meat production contributes significantly to climate change due to the emission of greenhouse gasses, including carbon dioxide, methane, and nitrous oxide [6].

A key strategy for achieving a sustainable food system is changing dietary behaviors [5]. Meat-based diets should, at least in part, be replaced by plant-based alternatives, such as legumes, or other protein sources, e.g., proteins derived from microalgae or fungi [7].

Among the promising approaches to meet the increasing global food demand is the consumption of edible insects. Insect farming offers several sustainability advantages over traditional livestock farming, including reduced land, water, and energy requirements, and lower greenhouse gas emissions [8]. In addition, some insect species contribute to a sustainable circular economy by utilizing organic waste as feed [9].

Insects are highly nutritious, providing proteins, minerals, and vitamins [10]. Furthermore, various insect-derived peptides exhibit beneficial biological activities, e.g., antioxidant, antimicrobial, anti-inflammatory, and antihypertensive effects [11]. However, the nutritional profile and biological properties vary significantly depending on a number of factors, including the species and developmental stage [12]. Despite these benefits, insect consumption may cause allergic reactions in some individuals, due to primary sensitization or cross-reactivity with allergens from other species, e.g., crustaceans or house dust mites [13].

Globally, over 2200 insect species are consumed across 128 countries [9]. However, in Europe, consumer acceptance of insects as food remains limited and is growing rather slowly [14]. To enhance acceptance, one strategy involves incorporating insects into non-recognizable forms, e.g., as powders, protein bars, and snacks [15,16].

Like all commercial food products, insect-based food must comply with legal regulations. In the European Union (EU), edible insects are regulated under the Novel Food regulation [17]. Novel Food is defined as food that had not significantly been consumed within the EU before 15 May 1997. To date, four insect species—the yellow mealworm (*Tenebrio molitor* larva), migratory locust (*Locusta migratoria*), house cricket (*Acheta domesticus*), and buffalo worm (*Alphitobius diaperinus* larva)—have been authorized for human consumption in the EU [18,19,20,21].

Food products must be safe and authentic to comply with food legislation. However, food adulteration remains a global concern. In the context of insect-based foodstuff, producers might attempt to increase profits by substituting higher-value insect species with less expensive ones. Palmer et al. demonstrated that different insect species exhibit varying degrees of cross-reactivity with shellfish tropomyosin [22]. Therefore, correct labeling of insect species may be important for consumers with insect allergies. More broadly, information about the presence of insects in food products is also relevant for vegetarians and vegans.

DNA-based methods are widely used for species identification and authentication [23]. Several polymerase chain reaction (PCR) assays have been published for the detection of specific insect species, including mealworm [24] and buffalo worm [25] in food products. Kim et al. introduced real-time PCR assays on a microfluidic chip for six edible insect species authorized for human consumption in Korea, including mealworm [26]. Köppel et al. developed a multiplex real-time PCR system to detect mealworm, migratory locust, and house cricket in food [27].

Recently, Hillinger et al. introduced a DNA metabarcoding assay to identify and differentiate edible insects in food [28]. DNA metabarcoding combines DNA barcoding with next-generation sequencing (NGS). DNA barcodes are DNA regions that are highly conserved at their ends, enabling amplification with universal primers. Sequence divergence between these conserved ends is required for species differentiation [29]. The universal primers designed by Hillinger et al. target a ~200 bp sequence of mitochondrial 16S rDNA. The DNA barcode is capable of distinguishing over 1000 insect species. The DNA metabarcoding assay, developed on Illumina platforms, allowed the detection of insects in highly processed and complex food products at proportions as low as 0.1% [28]. The assay has already been used to assess the rate of mislabeled insect-based products sold via EU e-commerce platforms [15].

In this study, we investigated whether the primer pair designed for DNA metabarcoding [28] could be applied for the identification and differentiation of the four EU-authorized edible insect species by DNA barcoding combined with high-resolution melting (HRM) analysis. HRM analysis involves amplifying the DNA barcode in the presence of a saturating DNA intercalating dye. The PCR products are then subjected to gradual heating, which causes DNA denaturation, release of the intercalating dye, and decrease in fluorescence intensity. The melting behavior of PCR products is influenced by various parameters, including the length and the guanine–cytosine content. HRM analysis is a very cost-effective high throughput method for the identification and differentiation of cultivars and closely related species [30]. It has been applied in various contexts, including the differentiation of *Hypericum* species in food supplements [31], authentication of *Gadidae* fish species [32], and differentiation of berry species [33].

## 2. Materials and Methods

### 2.1. Samples

Material from individual insect species (I1–I18, Table 1) was provided by the Institute for Sustainable Plant Production at the Austrian Agency for Health and Food Safety (AGES). The identity of the insect species was confirmed by experts from the Institute. Insect species I1–I4 have been authorized in the EU, while insect species I5-I18 have not yet been authorized in the EU. Insect-containing food products (S1–S20, Table 2) were purchased from supermarkets and online shops. All samples were stored at −20° C until DNA extraction was performed.

### 2.2. DNA Extraction

DNA was extracted from all samples by the cetyltrimethylammonium bromide (CTAB) method as described by Hillinger et al. [28]. In brief, samples were either cut into smaller pieces or homogenized in a mortar or lab mill. Sample lysis was performed in the presence of a CTAB/polyvinylpyrrolidone (PVP) extraction solution and proteinase K at elevated temperature under constant shaking. DNA was isolated using the Maxwell RSC Pure-Food GMO and Authentication Kit (Promega, Madison, WI, USA) and the Maxwell^®^ 16 instrument (Promega, Madison, WI, USA), following the manufacturer’s instructions. DNA concentration was determined photometrically at 260 nm using the QIAxpert spectrophotometer (Qiagen, Hilden, Germany). DNA extracts were stored at −20 °C.

### 2.3. PCR-HRM Analysis

Sequences of primers used in this study are provided in Table 3. Primers for insect species (Ins_f_, Ins_r_) were previously used for DNA metabarcoding [28]. Primers for house cricket (Hc_f_, Hc_r1_, Hc_r2_) and migratory locust (Ml_f1_, Ml_f2_) were designed during this study using PyroMark Assay Design Software 2.0.1.15 (Qiagen, Hilden, Germany). All primers were synthesized by Sigma-Aldrich (Steinheim, Germany) or TIB Molbiol (Berlin, Germany).

PCR-HRM analysis was performed on the Rotor-Gene Q instrument with a 72-well rotor (Qiagen, Hilden, Germany) using the Type-it HRM PCR Kit (Qiagen, Hilden, Germany). Each reaction was performed in a total volume of 20 µL, consisting of 18 µL PCR mix including EvaGreen, 2 mM MgCl_2_, the primers, and 2 µL DNA extract. DNA extracts with a DNA concentration > 2.5 ng/μL were diluted to 2.5 ng/µL, and DNA extracts with a lower DNA concentration were used undiluted. The PCR program was as follows: denaturation of double-stranded DNA; and activation of the polymerase: 95 °C, 5 min; amplification: 50 cycles; each cycle consisting of the following three steps: denaturation 94 °C, 15 s; annealing 58 °C, 30 s; elongation 72 °C, 30 s; final elongation: 72 °C, 10 min. Directly after final elongation, HRM analysis was performed, as follows: strand separation: 95 °C, 1 min; strand hybridization 40 °C, 1 min; HRM with a ramp from 65 °C to 95 °C with 0.1 °C/hold (2 s); and gain optimization (70% before melt).

Amplification and melt curves obtained by PCR-HRM were assessed using Rotor-Gene Q Series Software 2.3.1 (Qiagen, Hilden, Germany). Data were exported, analyzed, and presented graphically using OriginPro 2020 (OriginLab, Northampton, MA, USA).

### 2.4. Agarose Gel Electrophoresis

The identity of PCR products was assessed by gel electrophoresis (3% agarose (Sigma-Aldrich, Vienna, Austria) gel in 1 × TBE (Tris-borate-EDTA) buffer). The gel was post-stained with GelRed (Biotium, Fremont, CA, USA), and bands were visualized with a UVT-20 M transilluminator (Herolab, Wiesloch, Germany).

## 3. Results and Discussion

**Figure 1:**
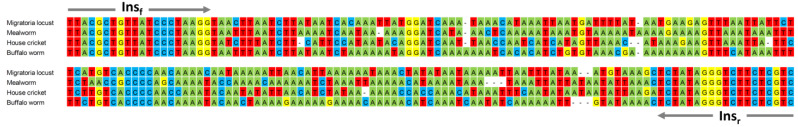
Alignment of the DNA barcode for migratory locust, mealworm, house cricket, and buffalo worm. Arrows indicate binding sites of insect primers Ins_f_ and Ins_r._

Our goal was to investigate if the primer pair designed for DNA metabarcoding [28] is applicable to discriminate between migratory locust, mealworm, house cricket, and buffalo worm, the four insect species authorized in the EU for human consumption, by DNA barcoding and HRM analysis. In principle, this should be possible, as indicated by the alignment of the DNA barcode (Figure 1).

The DNA barcode for migratory locust, mealworm, house cricket, and buffalo worm differ in length, the number of adenine, cytosine, guanine, and thymine, and the guanine-cytosine (GC) content (Table 4).

### 3.1. Adaptation of PCR Conditions

For PCR-HRM analysis, we commonly use the Type-it HRM PCR Kit (Qiagen, Hilden, Germany), containing the intercalating dye EvaGreen. Since its composition differs from that of the mastermix used in the study of Hillinger et al. [28], we started with adapting the PCR conditions. First of all, we adjusted the temperature protocol according to the recommendations of the provider of the Type-it HRM PCR Kit. When optimizing the MgCl_2_ concentration, the addition of 2 mM MgCl_2_ resulted in lower Ct values compared to the addition of 1 mM or 0 mM MgCl_2_. Thus, in all further experiments, 2 mM MgCl_2_ was added to the mastermix. Both the annealing temperature (58 °C) and the primer concentration (0.4 µM) used previously [28] turned out to be suitable and were applied without further optimization.

Under optimized conditions, DNA extracts from migratory locust, mealworm, and buffalo worm resulted in typical amplification curves. However, in the case of house cricket, the initial fluorescence signal was too high. In these experiments, all DNA extracts were diluted to a DNA concentration of 2.5 ng/µL. Due to the low DNA concentration (3.1 ng/µL) of the extract from house cricket, the dilution factor was drastically lower than that for the extracts from migratory locust, mealworm, and buffalo worm (DNA concentration ≥ 250.8 ng/µL). By diluting the DNA extract from house cricket 1:50, the initial fluorescence signal was as low as that obtained for the extracts from the other three insect species. Since the higher dilution factor did not have a negative impact on the amplification of the DNA barcode, in all further experiments the DNA extract from house cricket was diluted 1:50.

### 3.2. HRM Analysis of the Four Insect Species Authorized in the EU

**Figure 2:**
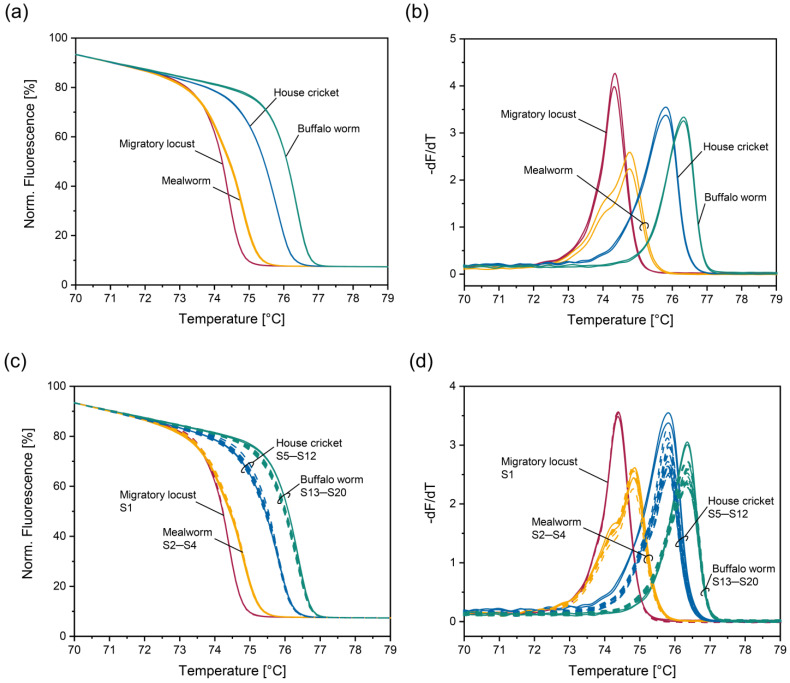
Normalized melt curves (**a**,**c**) and their negative derivative (**b**,**d**) obtained with the PCR-HRM assay involving insect primers Ins_f_ and Ins_r_. (**a**,**b**): Four edible insects authorized in the EU. (**c**,**d**) Commercial samples declared to contain EU-authorized edible insects. Dashed lines: positive controls; straight lines: commercial samples (S1–S20, Table 2). Extracts were analyzed in duplicates.

HRM analysis of the PCR products obtained for the four insect species authorized for human consumption in the EU resulted in the normalized melt curves and their negative derivative shown in Figure 2a and Figure 2b, respectively. Both the normalized melt curves and their negative derivative clearly indicate that the PCR products obtained for migratory locust, mealworm, house cricket, and buffalo worm differed in their melting behavior (Figure 2a,b). The peak maxima were at 74.3 °C, 74.8 °C, 75.7 °C, and 76.3 °C, respectively (Figure 2b). In the case of mealworm, a shoulder was observed at 74.0 °C (Figure 2b), hinting at an additional melt domain. The occurrence of two melting transitions was confirmed by Melting Curve Predictions Software uMelt Quartz (Release: 3.6.2 “Quartz”/Nov 5 2020) [34].

Next, we applied the PCR-HRM assay to twenty commercial food samples (S1–S20, Table 2) declared to contain migratory locust, mealworm, house cricket, or buffalo worm. Correct declaration of the food products with respect to insect species had been confirmed previously by DNA metabarcoding [28]. Normalized melt curves (Figure 2c) and their negative derivative (Figure 2d) overlapped with those obtained for the respective positive control, indicating that the food matrix did not have an impact on the melting behavior of the PCR products. Our findings suggest that the PCR-HRM assay is suitable for detecting the four insect species in highly processed products, such as crackers, bars, and chocolate down to a concentration of 2%.

### 3.3. Analysis of Insect Species That Have Not Been Authorized in the EU

Producers of insect-based food could be tempted to replace EU-authorized insect species with unauthorized ones. Thus, we investigated if the PCR-HRM assay allows discrimination between authorized and unauthorized insect species. In principle, the DNA barcode is suitable for distinguishing more than 1000 insect species [28]. However, although HRM analysis is highly applicable to detect sequence variations, its discrimination power is inherently lower compared to that of DNA metabarcoding.

We analyzed DNA extracts from 14 insect species (I5–I18, Table 1) that have not been authorized for human consumption in the EU. The black soldier fly (*Hermetia illucens*), terfly (*Musca domestica*), tropical house cricket (*Gryllodes sigillatus*), and silkworm (*Bombyx mori*) are among the insect species authorized for the production of processed animal protein intended for farmed animal feed in the EU [35,36]. This also applies to the Jamaican field cricket (*Gryllus assimilis*) [35]. However, a study by Weissman et al. [37] suggests that many European breeders who claim to sell *Gryllus assimilis* are actually selling *Gryllus locorojo* (banana cricket). We therefore included banana cricket in our study.

Extracts from fruit fliy and green bottle fly resulted in drastically higher Ct values (∆Ct > 10) and lower fluorescence signals compared to extracts from other insect species. For terfly, the DNA barcode could not be amplified repeatably. The DNA extract from banana cricket resulted in a too high initial fluorescence signal, as described above for house cricket. This problem could again be solved by diluting the extract 1:50.

**Figure 3:**
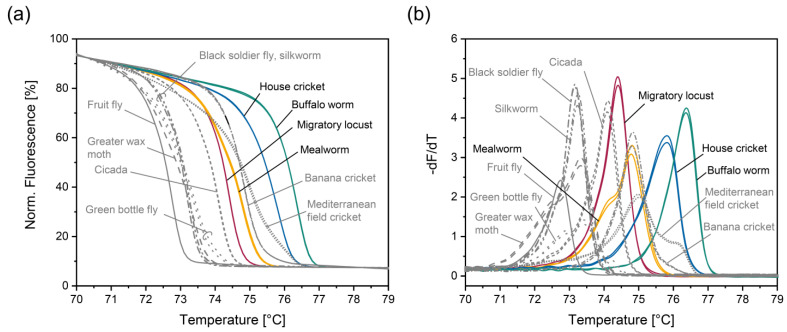

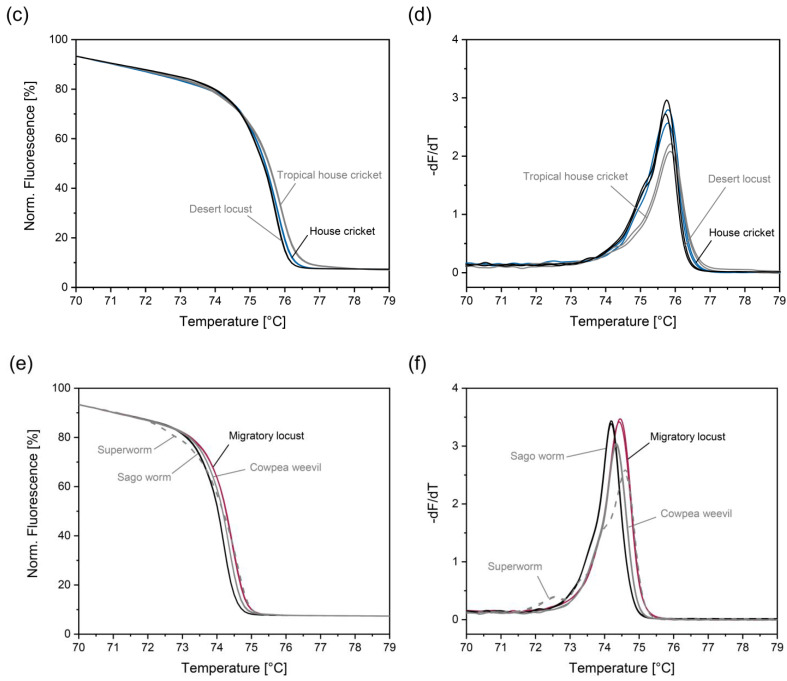
Normalized melt curves (**a**,**c**,**e**) and their negative derivative (**b**,**d**,**f**) obtained with the PCR-HRM assay involving insect primers Ins_f_ and Ins_r_. unauthorized insect species that (**a**,**b**) could be unambiguously distinguished from EU-authorized ones, (**c**,**d**) could not be unambiguously differentiated from house cricket, and (**e**,**f**) could not be unambiguously differentiated from migratory locust. Extracts were analyzed in duplicates.

Fruit fly, greater wax moth, cicada, great bottle fly, black soldier fly, silkworm, banana cricket, and Mediterranean field cricket resulted in normalized melt curves (Figure 3a) and their negative derivative (Figure 3b) that could clearly be differentiated from those obtained for EU-authorized insect species. Black soldier fly and silkworm led to similar normalized melt curves (Figure 3a) and their corresponding negative derivative (Figure 3b). Consequently, these two non-EU-authorized insect species could not be distinguished from each other.

Five non-EU-authorized insect species could not be unambiguously differentiated from EU-authorized ones. Tropical house cricket and desert locust resulted in similar normalized melt curves (Figure 3c) and corresponding negative derivatives (Figure 3d) as house cricket. PCR products for superworm, sago worm, and cowpea weevil showed similar melting behavior as that of migratory locust (Figure 3e,f). Thus, we tried to improve the discrimination power of the PCR-HRM assay by adding specific primers for house cricket and migratory locust, respectively.

### 3.4. Improving Discrimination of House Cricket from Tropical House Cricket and Desert Locust

In order to improve the differentiation of house cricket from the non-EU-authorized insect species tropical house cricket and desert locust, we designed specific primers for house cricket. Our aim was to obtain an additional PCR product for house cricket, resulting in a more complex melt curve compared to those for tropical house cricket and desert locust. Primers had to be compatible with the insect primers, Ins_f_ and Ins_r_, and should anneal at a temperature of ~58 °C.

**Figure 4:**
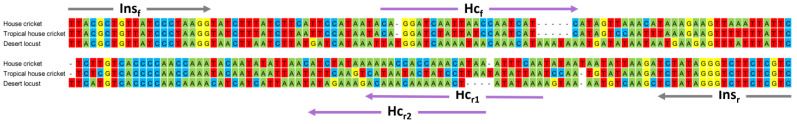
Alignment of the DNA barcode for the house cricket, tropical house cricket, and desert locust. Arrows indicate binding sites of insect primers, Ins_f_ and Ins_r_, and specific primers for house cricket, Hc_f_, Hcr_1_, and Hcr_2_.

The alignment of the DNA barcode for house cricket, tropical house cricket, and desert locust is shown in Figure 4. We designed one forward primer, Hc_f_, and two reverse primers, Hc_r1_ and Hc_r2_, for house cricket. The PCR product obtained with the primer pair Hc_f_ and Hc_r1_ was expected to be seven bp longer than that obtained with the primer pair Hc_f_ and Hc_r2_.

**Figure 5:**
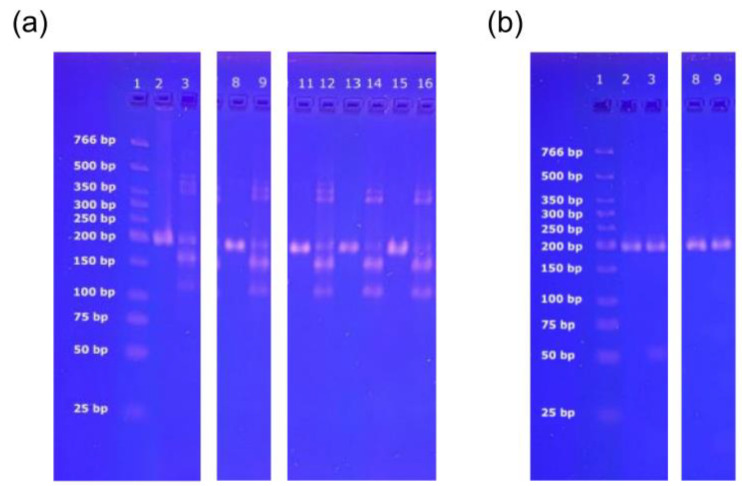
Pictures of agarose gels indicating the formation of additional PCR products with house cricket primer pair Hc_f_ and Hc_r1_. (**a**) Gel 1. lane 1: marker; lane 2, 3: house cricket; lane 8, 9: fusilli with cricket flour (S6, Table 2); lane 11, 12: seasoned crickets (tomato) (S8, Table 2); lane 13, 14: cricket cracker (rosemary, thyme) (S8, Table 2); lane 15, 16: cricket cracker (curcuma, smoked pepper) (S12, Table 2); lane 2, 8, 11, 13, 15: primers Ins_f_, Ins_r_; lane 3, 9, 12, 14, 16: primers Ins_f_, Ins_r_, Hc_f,_ Hc_r1_. (**b**) Gel 2. Lane 1, marker; lane 2, 3: tropical house cricket; lane 8, 9: desert locust; lane 2, 8: primers Ins_f_, Ins_r_; lane 3, 9: primers Ins_f_, Ins_r_, Hc_f_, Hc_r1_.

The addition of house cricket-specific primers Hc_f_ and Hc_r1_ actually resulted in the formation of additional PCR products for house cricket, as indicated by the band patterns obtained by agarose gel electrophoresis (Figure 5a). The bands hint at the formation of PCR products of ~150 bp (i.e., 152 bp long product by primers Hc_f_ and Ins_r_) and ~120 bp (i.e., 117 bp long product by primers Hc_f_ and Hc_r1_). In addition, slight bands suggest the formation of products of ~350 bp. Additional PCR products were only obtained for house cricket (lane 3, Figure 5a) and food products declared to contain house cricket (lane 9, 12, 14, 16, Figure 5a), but neither for tropical house cricket (lane 3, Figure 5b) nor for desert locust (lane 9, Figure 5b).

**Figure 6:**
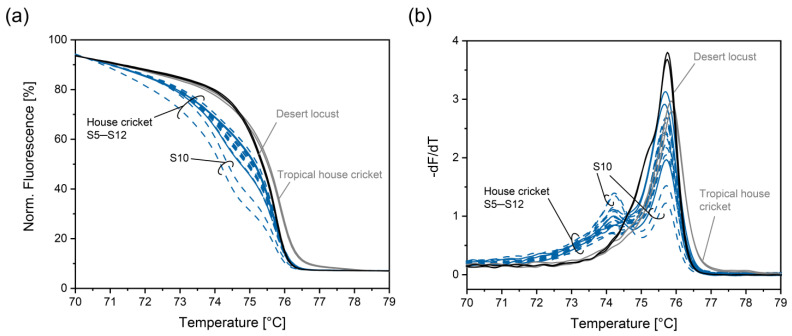
Normalized melt curves (**a**) and their negative derivative (**b**) obtained with the PCR-HRM assay involving insect primers, Ins_f_ and Ins_r_, and house cricket primers, Hc_f_ and Hc_r1_, for the house-cricket, desert locust, and tropical house cricket. Extracts were analyzed in duplicates.

By the formation of additional PCR products, the normalized melting curve for house cricket was altered (Figure 6a), increasing discrimination from tropical house cricket and desert locust. In the negative derivative, an additional peak at ~74.2 °C was observed (Figure 6b). For most samples declared to contain house cricket, normalized melt curves were similar to those for the positive control. However, in the case of S10 (cricket cracker (tomato, oregano)), normalized melt curves deviated and were also less repeatable.

The primer pair Hc_f_/Hc_r2_ also resulted in the formation of additional PCR products and consequently altered the melt curve for house cricket, without having an impact on the melt curves for tropical house cricket and desert locust. However, since this primer pair resulted in lower fluorescence intensities, all further experiments were performed with primer pair Hc_f_/Hc_r1_.

### 3.5. Improving Discrimination of Migratory Locust from Superworm, Cowpea Weevil, and Sago Worm

**Figure 7:**
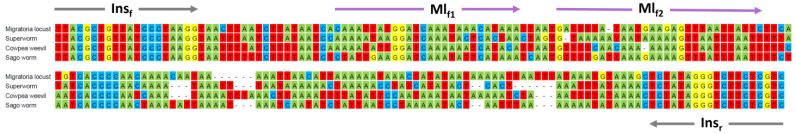
Alignment of the DNA barcode for the migratory locust, superworm, cowpea weevil, and sago worm, and binding sites of insect primers, Ins_f_ and Ins_r_, and primers specific for migratory locust, Ml_f1_ and Ml_f2_.

We tried to improve the discrimination of migratory locust from superworm, cowpea weevil, and sago worm by applying the same strategy as described for house cricket and designing specific primers for migratory locust. However, in order to keep the number of primers in the reaction mix as low as possible, we refrained from the design of reverse primers and only designed forward primers. Figure 7 shows the alignment of the DNA barcode for the four insect species and the primer binding sites of the insect primers, Ins_f_ and Ins_r_, and two forward primers specific for migratory locust, Ml_f1_ and Ml_f2_.

**Figure 8:**
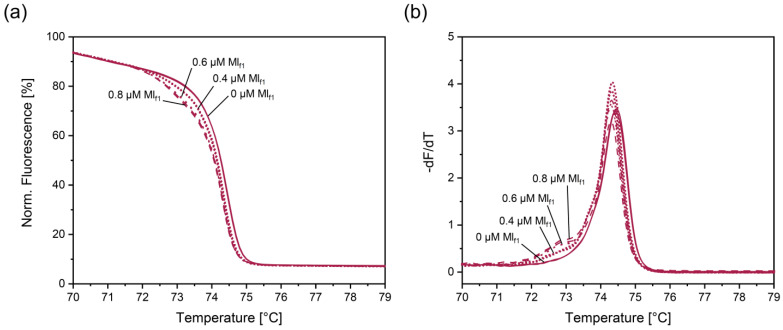
Impact of the Ml_f1_ concentration on normalized melt curves (**a**) and their negative derivative (**b**) for the migratory locust, obtained with the PCR-HRM assay involving insect primers, Ins_f_ and Ins_r_, and the migratory locust primer, Ml_f1_.

Among the two primers, only Ml_f1_ led to a small shift in the normalized melting curve for migratory locust. Since 0.8 µM Ml_f1_ resulted in a more pronounced shift (Figure 8a) and a higher additional peak at 72.8 °C (Figure 8b) than 0.4 μM and 0.6 μM Ml_f1_, a concentration of 0.8 µM Ml_f1_ was used in all further experiments.

### 3.6. Optimized PCR-HRM Assay Involving Primers Ins_f_, Ins_r_, Hc_f_, Hc_r1_, and Ml_f1_

The optimized PCR-HRM assay involved five primers: 0.4 µM of the insect primers, Ins_f_ and Ins_r_, designed for DNA metabarcoding [28], 0.4 µM of the house cricket primers, Hc_f_ and Hc_r1_, and 0.8 μM of the migratory locust primer, Ml_f1_.

**Figure 9:**
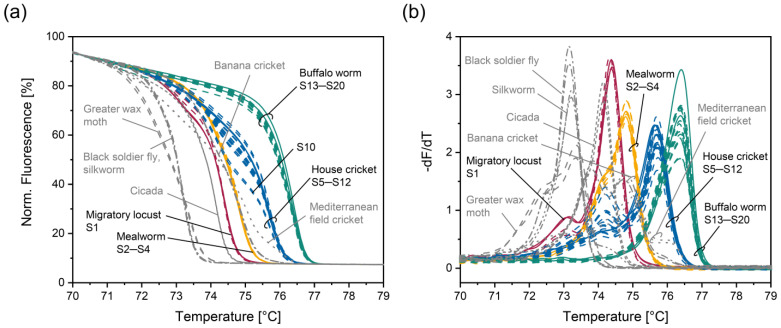

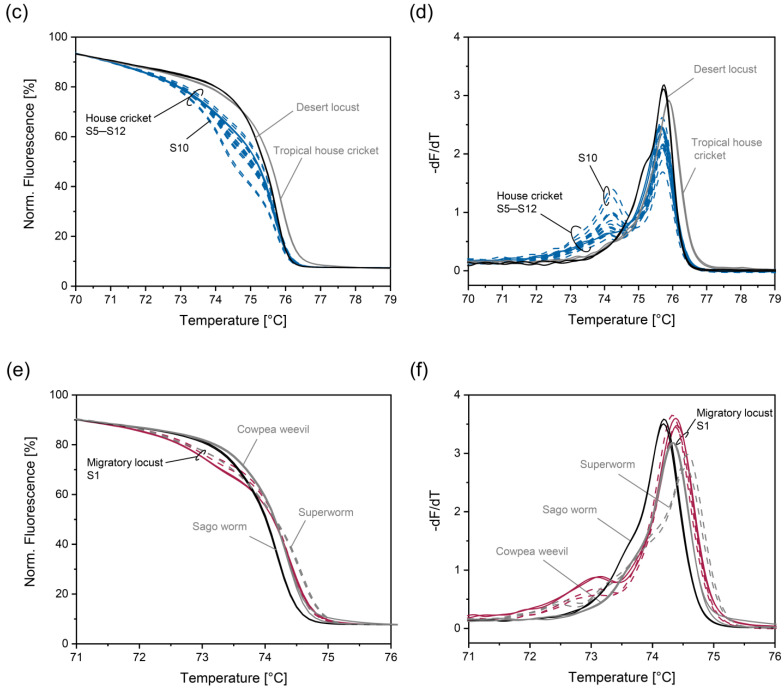
Normalized melt curves (**a**,**c**,**e**) and their negative derivative (**b**,**d**,**f**) obtained with the PCR-HRM assay involving insect primers, Ins_f_ and Ins_r_, house cricket primers, Hc_f_ and Hc_r1_, and migratory locust primer, Ml_f1_. (**a**,**b**): Four edible insects authorized in the EU, commercial samples declared to contain EU-authorized edible insects, and non-EU-authorized insect species that could be distinguished with the PCR-HRM assay involving insect primers, Ins_f_ and Ins_r_. (**c**,**d**): House cricket, desert locust, and tropical house cricket. (**e**,**f**): Migratory locust, superworm, cowpea weevil, and sago worm. Extracts were analyzed in duplicates.

Alterations in the normalized melt curves and their negative derivative, caused by the addition of specific primers for house cricket and migratory locust, did not hamper the discrimination between the four EU-authorized insect species (Figure 9a,b). Normalized melt curves and their negative derivative for commercial samples (Figure 9a,b) deviated more from the respective positive controls, compared to the PCR-HRM assay involving only the two insect primers (Figure 2c,d). However, the insect species contained in the food could be identified correctly.

The optimized PCR-HRM assay did not lead to PCR products for fruit fly, green bottle fly, and terfly. The four EU-authorized insect species could unambiguously be distinguished from the eleven unauthorized insect species for which PCR products were obtained (Figure 9a–f).

### 3.7. Strengths and Limitations of the Optimized PCR-HRM Assay

Previous studies have developed real-time PCR methods for detecting insect species in food, relying on species-specific primers and probes. For example, real-time PCR assays have been reported for mealworm [24] and buffalo worm [25]. Köppel et al. [27] extended this approach by combining three species-specific systems—for the mealworm, migratory locust, and house cricket—to a multiplex real-time PCR assay.

In contrast, the PCR-HRM assay developed in this study does not require species-specific probes. It differentiates insect species based on differences in the melting behavior of PCR products obtained by amplifying a ~200 bp fragment of mitochondrial 16S rDNA. This untargeted approach enables the detection of unexpected species that might be overlooked by targeted methods such as real-time PCR.

Compared to DNA metabarcoding, which combines DNA barcoding with NGS, the PCR-HRM has lower discrimination power. However, HRM analysis offers significant advantages in terms of time and labor efficiency. Unlike DNA metabarcoding, it does not require DNA library preparation, sequencing, and complex bioinformatic analysis, allowing for much faster results. While metabarcoding is cost-effective for analyzing large sample sets or multiple parameters [38], PCR-HRM is more economical on a per-sample basis. The cost of PCR-HRM is approximately 1.50 Euros per sample, whereas amplicon sequencing is about 25 times more expensive. Thus, the optimized PCR-HRM assay serves as a screening tool for authenticating EU-authorized edible insect species in food.

Currently, eight insect species are authorized for use in processed animal protein intended for farmed animal feed in the EU [35,36], including the black soldier fly, terfly, yellow mealworm, buffalo worm, house cricket, tropical house cricket, Jamaican field cricket, and silkworm. However, the PCR-HRM assay presented in this study is not suitable for verifying EU-authorized insect species in feed, since it does not enable differentiation between black soldier fly and silkworm. In addition, the DNA barcode for the terfly could not be amplified repeatedly, limiting its applicability for this species.

## 4. Conclusions

The insect primers previously designed for DNA metabarcoding, targeting a ~200 bp sequence of mitochondrial 16S rDNA, turned out to be suitable for developing a PCR-HRM assay for discrimination between the four EU-authorized edible insect species in food. The PCR-HRM assay allowed unambiguous identification of the four insect species in highly processed food samples, including crackers, bars, and chocolate.

However, several unauthorized insect species resulted in similar melt curves as the authorized ones. Specifically, desert locust and tropical house cricket could not be distinguished from house cricket, and PCR products obtained for superworm, cowpea weevil, and sago worm showed similar melting behavior as the PCR product for migratory locust.

The selectivity of the PCR-HRM assay could be improved by designing specific primers for house cricket and migratory locust, respectively. With the formation of additional PCR products, the normalized melting curves for house cricket and migratory locust were altered, increasing discrimination from the non-EU-approved insect species.

The optimized PCR-HRM assay included five primers, the insect primers, Ins_f_ and Ins_r_, the house cricket primers, Hc_f_ and Hcr_1_, and the migratory locust primer, Ml_f1_. Although normalized melt curves and their negative derivative obtained for commercial samples deviated more from the respective positive controls, compared to the PCR-HRM assay involving the two insect primers, the insect species could be identified unambiguously.

Compared to metabarcoding, the analysis of DNA barcodes by HRM is very cost-efficient. Thus, the optimized PCR-HRM assay can be applied as a screening tool for authentication of EU-authorized edible insect species in food.

## Figures and Tables

**Table 1:** Insect species analyzed. I1–I4: species authorized in the EU; I5–I18: species not authorized in the EU.

Insect Species ID	Scientific Name	Commercial Name
I1	*Locusta migratoria*	migratory locust
I2	*Tenebrio molitor*	mealworm
I3	*Acheta domesticus*	house cricket
I4	*Alphitobius diaperinus*	buffalo worm
I5	*Drosophila hydei*	fruit fly
I6	*Galleria mellonella*	greater wax moth
I7	*Lucilia sericata*	green bottle fly
I8	*Hermetia illucens*	black soldier fly
I9	*Bombyx mori*	silkworm moth
I10	*Cicadae*	cicada
I11	*Rhynchophorus ferrugineus*	sago worm
I12	*Zophobas atratus*	superworm
I13	*Callosobruchus maculatus*	cowpea weevil
I14	*Musca domestica*	terfly
I15	*Gryllus bimaculatus*	Mediterranean field cricket
I16	*Gryllus locorojo*	banana cricket
I17	*Gryllodes sigillatus*	tropical house cricket
I18	*Schistocerca gregaria*	desert locust

**Table 2:** Commercial insect-based food products. The labels of products S1, S3, S4, S8–S10, S12, S14, and S16–S18 also included the scientific name of the insect species.

Sample ID	Product	Insect Species Declared ^1^
S1	locust, blanched, freeze-dried	migratory locust
S2	mealworms	mealworm
S3	dark chocolate with roasted mealworms	mealworm (2%)
S4	whole milk chocolate with roasted mealworms	mealworm (2%)
S5	crickets	cricket ^2^
S6	fusilli with cricket flour	cricket ^2^
S7	fried crickets	cricket ^2^
S8	seasoned crickets (tomato)	house cricket
S9	seasoned crickets (smoked)	house cricket
S10	cricket crackers (tomato, oregano)	house cricket (15%)
S11	cricket crackers (rosemary, thyme)	house cricket (16%)
S12	cricket cracker (curcuma, smoked pepper)	house cricket (15%)
S13	ready-to-mix beetroot risotto with insect protein	buffalo worm (5.7%)
S14	ready-to-mix brownie cake with insect protein	buffalo worm meal (5.5%)
S15	ready-to-mix oat patty with insect protein	buffalo worm (12%)
S16	raw bar sour cherry with insect protein	buffalo worm (12%)
S17	raw bar apple strudel with insect protein	buffalo worm (12%)
S18	raw bar apricot with insect protein	buffalo worm (13%)
S19	protein shake with buffalo worm (strawberry flavor)	buffalo worm (50%)
S20	peanut cream with buffalo worm	buffalo worm (17%)

^1^ Correct declaration of all products had been confirmed by DNA metabarcoding [28]; ^2^ Identified as house cricket by DNA metabarcoding [28].

**Table 3:** Primer sequences. Ins: insects; Hc: house cricket; Ml: migratory locust; f: forward; r: reverse.

Primer ID	Sequence (5′ → 3′)	Target Species	Reference
Ins_f_	TWACGCTGTTATCCCTAAGG	insects	[28]
Ins_r_	GACGAGAAGACCCTATAGA	insects	[28]
Hc_f_	CAGGATCAATTAACCAATCATC	house cricket	this work
Hc_r1_	TTGAAATTTATGTTTGGTGGTTTT	house cricket	this work
Hc_r2_	TTATGTTTGGTGGTTTTTTATAGAT	house cricket	this work
Ml_f1_	CAAATTATGGATCAAATAAACATAAA	migratory locust	this work
Ml_f2_	GATTTTATAATGAAGAGTTTAATTATTC	migratory locust	this work

**Table 4:** Characteristics of the DNA barcode for migratory locust, mealworm, house cricket, and buffalo worm. bp: base pairs; A: adenine; C: cytosine; G: guanine; T: thymine.

Insect Species	Length [bp]	Number of Bases				GC Content [%]
		A	C	G	T	
migratory locust	198	86	28	17	67	22.7
mealworm	197	98	29	15	55	22.3
house cricket	196	77	40	15	64	28.1
buffalo worm	198	93	34	18	53	26.3

## Data Availability

The original contributions presented in this study are included in the article. Further inquiries can be directed to the corresponding author.
